# Efficacy of a smartphone-based coaching program for addiction prevention among apprentices: study protocol of a cluster-randomised controlled trial

**DOI:** 10.1186/s12889-020-09995-6

**Published:** 2020-12-14

**Authors:** Severin Haug, Raquel Paz Castro, Andreas Wenger, Michael P. Schaub

**Affiliations:** Swiss Research Institute for Public Health and Addiction at Zurich University, Konradstrasse 32, 8005 Zurich, Switzerland

**Keywords:** Addiction, Prevention, Apprentices, Coaching, Smartphone

## Abstract

**Background:**

A large proportion of apprentices shows addictive behaviours like cigarette smoking, alcohol, cannabis, or compulsive Internet use, others do not show such behaviours at all. *ready4life* is a smartphone application-based coaching program for apprentices, which takes into account the heterogeneity of adolescent addictive behaviour by promoting life skills and reducing risk behaviours. The main objective of the planned study is to test the efficacy of *ready4life* for addiction prevention among apprentices in Switzerland within a controlled trial.

**Methods/design:**

The efficacy of the *ready4life* coaching program will be tested in comparison to an assessment only control group, within a cluster-randomised controlled trial with one follow-up assessment after 6 months. At the beginning of the program, participants of the intervention group will receive an individual profile, showing areas in which they have sufficient resources and in which there is a need for coaching. Based on this feedback, they can select two out of the following six program modules: stress, social skills, Internet use, tobacco/e-cigarettes, cannabis, and alcohol. Participants of the intervention group will receive individualised coaching by a conversational agent (chatbot) for a period of four months. The coaching relies on motivational and social-cognitive principles of behaviour change. Within weekly dialogues, the coach provides individually tailored information in different formats, such as videoclips, texts, or pictures. Study participants will be 1318 apprentices with a minimum age of 15, recruited in approximately 100 vocational school classes in Switzerland. Primary outcome will be a composite measure for addictive behaviours including (1) at risk-drinking, (2) tobacco/e-cigarette smoking, (3) cannabis use, and (4) problematic Internet use.

**Discussion:**

The study will reveal whether this universally implementable but individually tailored intervention approach is effective in preventing the onset and escalation of addictive behaviors among apprentices.

**Trial registration:**

ISRCTN59908406 (registration date: 21/10/2020).

## Background

Adolescence is characterized by several biological, psychological and social changes, which have a significant impact on the further course of life [1, 2]. These changes enable greater autonomy, the building of social relationships as well as physical and social identity. However, they are also accompanied by an increased willingness to take risks during a time when the cognitive functions of the brain, e.g. to regulate emotions, are not yet fully developed [3]. This also increases the susceptibility to psychological and substance use disorders [4].

For adolescents starting an apprenticeship, entering work life is accompanied by additional changes and new challenges. Financial independence and social detachment from their parents result in a higher degree of autonomy. At the same time, the responsibility for one’s own actions increases and the workplace environment confronts them with the reality of work, which is often accompanied by stress, time and success pressure. Dealing with colleagues, superiors and business partners or customers also places new demands on the social competence of the apprentices. Accordingly, the beginning of the apprenticeship is associated with health risks, especially increased substance consumption. Although corresponding comparative figures from Switzerland are not available, the German drug affinity study shows a significantly higher proportion of tobacco smokers among apprentices than among high school students of the same age (36% vs. 19%), whereas the difference is less pronounced for binge drinking, an indicator of problematic alcohol consumption (43% vs. 39%) [5]. Apprentices, typically in the age of 15–18 years, represent a very heterogeneous group in terms of protective and risk behaviours, [6, 7] with a smaller proportion showing no substance use or addictive behaviours and another showing at least one or co-occurring at-risk behaviours. For example in vocational school students from Switzerland, 81% of smokers showed at-risk alcohol use whereas only 49% of non-smokers drink hazardously [7].

According to international reviews, general life-skills training programs that address social skills and simultaneously address social influences, e.g. by media or the peer group, are particularly suitable for preventing substance use or delaying the onset of addictive behaviours in younger adolescents and those who have not yet started using addictive substances [8]. In adolescents already using substances, interventions based on motivational and cognitive-behavioural principles proved to be effective in reducing addictive behaviours. They are effective at preventing the onset of specific substance use [9–11] or at decreasing problematic substance use [12]. Due to their age group and their heterogeneous risk profile ranging from none to several co-occurring addictive behaviors, both general life-skills training programs and motivational, cognitive-behavioral programs to reduce specific substances show potential.

Although schools provide access to large numbers of students, the implementation and dissemination of general life-skills or addiction prevention programs in schools pose serious challenges [13]. First, teachers and other professionals need the time, motivation, knowledge and skills to deliver the program. Second, extensive resources in terms of personnel, money, and time are required to administer such programs.

Digital intervention programs, e.g. delivered via computer, Internet or mobile phone have the potential to overcome the above-mentioned obstacles that hinder successful program implementation and larger-scale dissemination of preventive intervention programs in schools. They have a wide reach at a low cost and offer the opportunity to automatically deliver contents that can be accessed at any time and in any place [14, 15]. Furthermore, they allow an individual tailoring of the intervention program according to the individual health risks, resources and preferences. A meta-analysis on school-based eHealth interventions targeting multiple lifestyle risk behaviours [16] retrieved small and short-term intervention effects regarding diet, physical activity and screen time, but no effects for alcohol use or smoking behavior, however, no studies of mobile health (mHealth) intervention programs were included.

A promising means of delivering prevention programs is to do so remotely by mobile technologies. In Switzerland, as in most other developed countries, 99% adolescents between the ages of 12 and 19 own a mobile phone [17], rendering such programs universally implementable. Most adolescents use mobile phones on a daily basis for texting, taking pictures, playing games etc. Mobile phone-based interventions can provide almost constant support to users, in comparison to interventions that can only be accessed at specific times or locations. Mobile phone-based interventions also provide a discrete and confidential means of intervention delivery [18].

Although no mobile phone-delivered interventions addressing multiple lifestyle risk behaviours have been tested yet, mHealth interventions addressing single risk behaviours or general life-skills are promising [6, 19–21]. A controlled trial on a text messaging-based intervention to reduce problem drinking in vocational and upper secondary school students in Switzerland showed a significant intervention effect on the prevalence of binge drinking. For example binge drinking decreased by 6% in the intervention group and increased by 3% in the control group, relative to that of baseline assessment [19].

Another Swiss study showed the acceptance and potential effectiveness of a mobile phone-based life-skills training program for substance use prevention among non-smoking vocational school students [6]. Pre-post comparisons revealed a decrease in perceived stress and increases in several life skills addressed between baseline and the follow-up assessment. The proportion of adolescents with at-risk alcohol use significantly declined from 20% at baseline to 15% at follow-up.

Based on the promising effectiveness of mobile phone-delivered life-skills and addiction prevention programs among vocational school students and the heterogeneous addictive behaviour in this target group of late-adolescents, a reasonable next step is to test the efficacy of a program combining life-skills training and the prevention of multiple specific addictive behaviours within a controlled trial.

Within this study protocol, we describe a cluster randomised controlled trial testing the efficacy of a combined life-skills and addiction prevention coaching program delivered via smartphone to prevent addictive behaviours among apprentices recruited in vocational schools.

## Methods/design

### Design and hypotheses

**Fig. 1:**
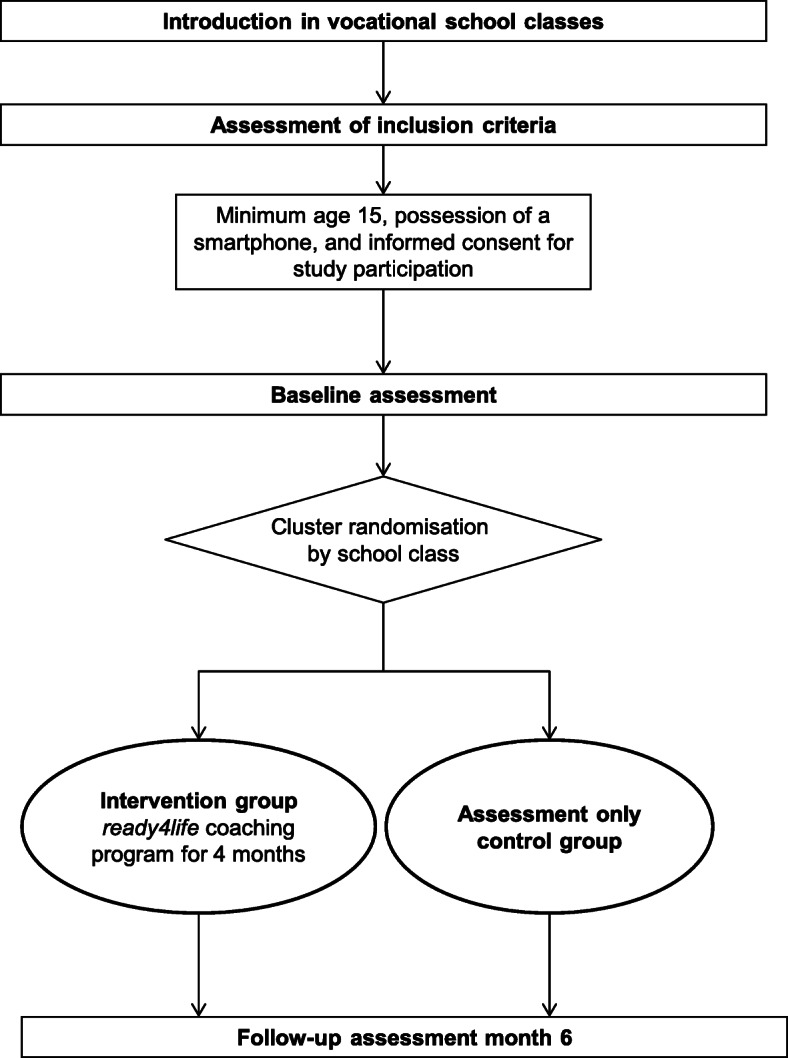
Study design

We will conduct a two-arm cluster-randomised controlled trial to test the efficacy of *ready4life*, a smartphone-based life-skills and addiction prevention coaching program to prevent substance use among apprentices. We will test the efficacy of the intervention in comparison to an assessment only control group. The study participants will be assessed at baseline and at 6-months follow-up (Fig. 1). Our main hypothesis is that the individually tailored 4-month intervention program will be more effective than assessment only, in preventing the onset and escalation of addictive behaviours including (1) at risk-drinking, (2) tobacco/e-cigarette smoking, (3) cannabis use, and (4) problematic Internet use at 6-months follow-up.

### Participants, setting and procedure

In most European countries, vocational schools are post-secondary public schools that are analogous to American community colleges. They are part of the dual educational system that combines apprenticeships in a business context and vocational training in a school context. Vocational schools provide general education and specific skills for each particular profession.

Based on data from the Swiss Federal Statistical Office, approximately half of all Swiss adolescents ages 16 to 19 currently attend vocational schools [22], with the highest proportions among adolescents ages 17 (males: 60%, females: 47%) and 18 (males: 57%, females: 45%). Vocational schools in the German speaking part of Switzerland will be invited to participate in the study by cooperating regional centres for addiction prevention.

Prevention specialists from these regional centres for addiction prevention will be trained and informed on the ready4life intervention program. These specialists will arrange sessions in the participating vocational school classes, lasting 30 min during regular school lessons reserved for health education. Within this session, the students will be informed about the program and invited to participate in a study testing innovative channels for the delivery of health-related information and life skills. The students will be informed about the study’s aims, design, assessments, reimbursement, and data protection. The chatbot coaching program, its friendly competition and the chance to win prizes will be described in detail, using a teaser video (https://www.r4l.swiss/). To ensure sufficient participation and thus representativeness of the sample [23], students will be informed that they would also receive a small reward for participating in the study (10 CHF for completing the follow-up assessment). Participating students will download the app on their smartphone and complete online study registration and baseline assessment. After giving informed consent, study participants will be invited to choose a username and provide their mobile phone number.

Subsequently, participants of intervention classes will receive an individual profile on their risks and resources and can select two topics (stress, social skills, Internet use, tobacco, cannabis, and alcohol) on which they will receive coaching, for a period of four months (two months per topic) by the chatbot. Participants of the assessment only control classes will not receive the intervention program. However, they will be invited to participate in the program after completion of the follow-up assessment in month 6. Follow-up assessment will be conducted in both study groups using a similar procedure: participants will be invited to complete the online follow-up assessments via SMS text messaging. Non-responders will additionally be addressed via computer-assisted telephone interviews conducted by research assistants.

### Ethical review

The study protocol was approved by the the Ethics Committee of the Faculty of Arts and Sciences at the University of Zurich (approval number 20.10.12; date of approval October, 16th, 2020). The trial will be executed in compliance with the Helsinki Declaration.

### Randomisation and allocation concealment

To avoid spill-over effects within school classes, we will conduct a cluster-randomised controlled trial using school class as a randomisation unit. Due to the heterogeneity of students in the different vocational schools, we will use a separate randomisation list for each school (stratified randomisation). Furthermore, to approximate equality of sample sizes in the study groups, we will use block randomisation with computer generated randomly permuted blocks of 4 cases [24].

The prevention specialists supervising the baseline assessment will be blinded to the group allocation of school classes. In addition, group allocation will not be revealed to participants until they had provided their informed consent, username, mobile phone number, and baseline data. Furthermore, the research assistants who perform the computer-assisted follow up assessments for primary and secondary outcomes will be blinded to the group allocation.

### Sample size calculation

Based on reviews on electronically-delivered programs for the prevention of addictive disorders [25–29], we expect a small effect size for the main outcome of this study, the non-parametric composite measure of addictive behaviours. Based on an expected Cohen’s d of 0.2, a sample size of *n* = 412 in each study group would be required to have 80% power for a Wilcoxon-Mann-Whitney-test (α = 5%, 2-sided) in order to detect this difference based on a calculation using G-Power. As vocational school students are nested within school classes, we additionally need to consider a potential design effect for the calculation of the sample size for our study. Based on previous efficacy studies in vocational schools [6, 19], an average cluster size of 13 study participants per school class and an intra-cluster correlation coefficient of 0.05 could be expected. This would result in a design effect of 1.60. Multiplying this design effect by the required size for an unnested sample (n = 412) results in a required sample size of *n* = 659 per study group and a total of *n* = 1318 study participants. Thus, based on the participation rates of previous efficacy studies among vocational school students [19, 21], approximately 100 school classes are required to reach this sample size.

### Intervention program

**Fig. 2:**
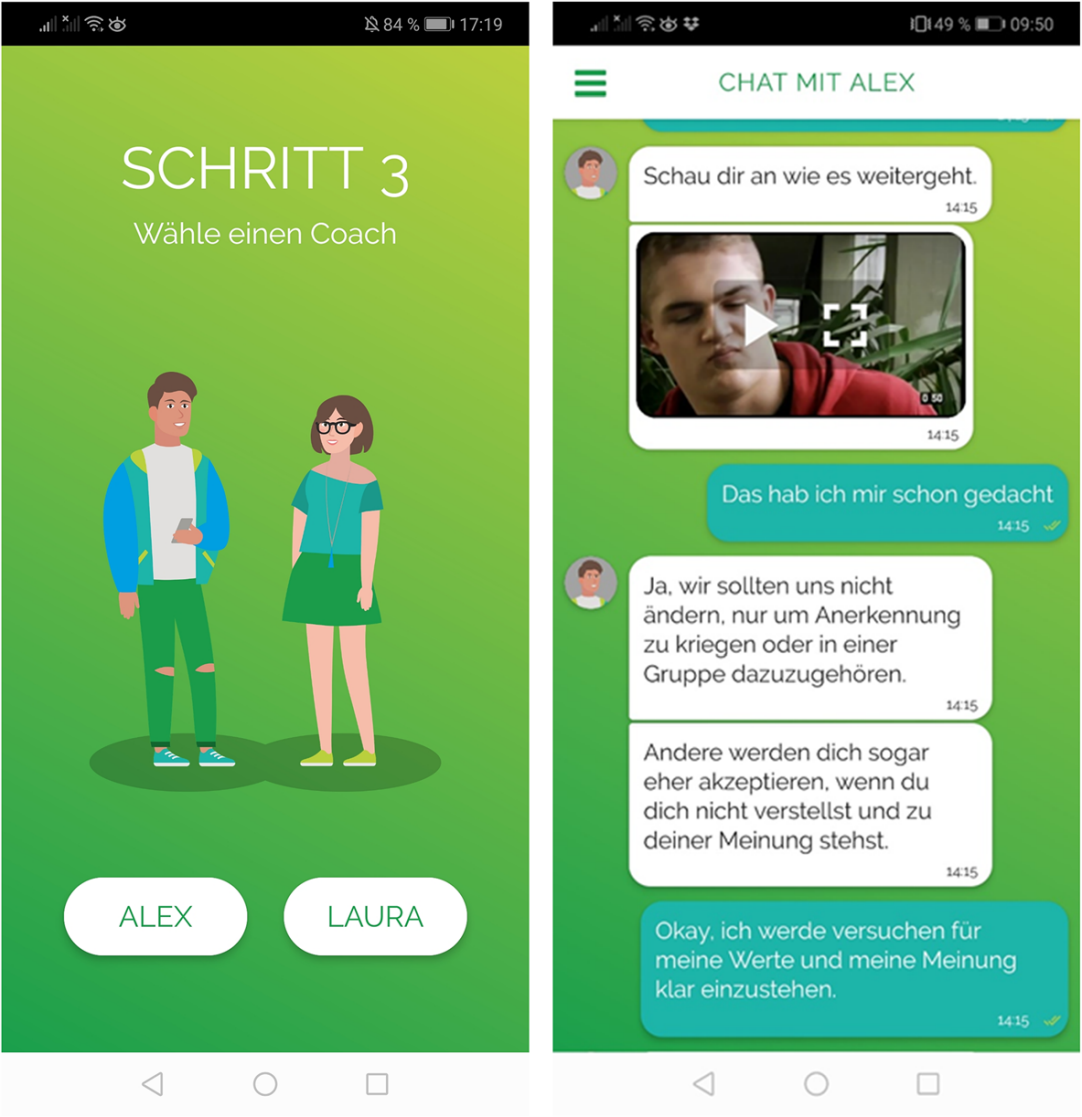
Screenshots from the *ready4life* coaching program (left: selection of avatar, right: sample chatbot dialogue)

*ready4life* (www.r4l.swiss) is a smartphone application-based addiction prevention program for adolescents that takes into account the heterogeneity of adolescent addictive behaviour by promoting life skills on the one hand and reducing risk behaviours on the other. The program provides individually tailored coaching by a conversational agent (chatbot, see Fig. 2).

After choosing a male or female avatar, participants are invited by the chatbot to provide demographic data (age and sex) and to complete the baseline assessment on stress, self-efficacy, social skills, Internet, tobacco/e-cigarette, cannabis, and alcohol use. Subsequently, an individual feedback is generated on the basis of this survey conducted via smartphone. Using the traffic light colours, this feedback shows areas in which a participant has sufficient resources and in which there is a need for coaching or counselling. Based on this feedback, participants can select two out of the following six program modules: stress, social skills, Internet use, tobacco/e-cigarettes, cannabis, and alcohol. Using the individual data from the baseline assessment, participants receive coaching for a period of 8 weeks for each of the two selected topics. During this 4-month coaching the virtual coach motivates the participants to deal sensibly with addictive substances, gives feedback on current consumption and life skills, and provides individually tailored information in weekly dialogues. Participants are notified of a new module every Tuesday via push message. The time required to process a weekly dialog is between two and five minutes. Participants can also start a conversation with the chatbot themselves by selecting one of several predefined topics within each module (e.g. “quiz on myths about alcohol” within the alcohol module or “using body language effectively” within the module on social skills). In a separate chat within the app (“Ask the Expert”) the participants can pose personal questions to regional addiction prevention experts. Young people with high-risk addictive behaviour are encouraged to seek further advice (e.g., regional outpatient counselling services). In order to stimulate active program engagement, several interactive elements such as quiz questions, contests and a playful competition are integrated into *ready4life*. Program users can collect credits for each completed weekly dialogue with the chatbot. The more credits participants will collect, the higher their chances will be of winning one of several attractive prizes which will be part of a prize draw after program completion. Participants will be able to retrieve their number of credits compared to the number of credits of other program participants’ of their group (similar starting date) at any time from an individual profile page.

Each program module has a similar structure (Table 1) and contains similar intervention elements based on principles of the Social-Cognitive Theory (e.g., goal-setting, self-monitoring, observational learning) [30], the Social Norms Approach (e.g., normative feedback) [31] and Motivational Interviewing (e.g., decisional balance) [32].

**Table 1:** Coaching contents and elements of ready4life: similar structure for each of the six modules on stress, social skills, Internet use, tobacco/e-cigarettes, cannabis, and alcohol

Week	Objective	Example: tobacco/e-cigarettes	Scientific principle
1	Getting individual feedback	Feedback on smoking costs Smoking prevalence in reference group	Self-Monitoring Normative Feedback
2	Realizing risks Motivation to improve skills	Pros and cons of smoking and smoking cessation	Decisional balance Outcome expectations
3	Learning from other participants	Picture Contest “What could help you or others to become smoke-free?”	Observational Learning Facilitation
4	Creating if-then behavior plan	If-then-plan for dealing with temptation in smoking situations	Implementation Intention Self-Regulation
5	Clarifying individual questions	Possibility to ask an expert on smoking and smoking cessation	Facilitation
6	Selecting and pursuing a personal goal	Personal challenge on the subject of smoking	Goal-setting Self-Monitoring
7	Learning to deal with difficult situations	Video quiz on the social norm of smoking in children Motivation to resist peer pressure	Observational Learning Self-Efficacy
8	Broadening one’s own perspective	Summary of frequently asked questions and expert answers	Observational Learning Self-Efficacy

### Assessments and outcomes

At baseline, we will assess individual demographic variables (age, sex) as well as characteristics of the schools and school classes (type of vocational school, class size, apprenticeship profession, number of students present within school class). Baseline- and follow-up assessment will include the following addictive behaviours and life skills addressed in the intervention program:

1. At risk-drinking in the preceding 30 days, according to guidelines of the Swiss Federal Office of Public Health [33].
2. 30-days point prevalence for tobacco/e-cigarette smoking, according to the criteria of the Society for Nicotine and Tobacco Research [34].
3. 30-days point prevalence for cannabis use.
4. Problematic Internet use assessed by the Short Compulsive Internet Use Scale [35].
5. General self-efficacy assessed by the Short Scale for Measuring General Self-efficacy Beliefs [36].
6. Stress assessed by a single-item measure of stress symptoms [37].

The primary outcome of the planned study is a composite measure (range: 0–4) for addictive behaviours, reflecting the number of risk behaviours (at-risk drinking, tobacco/e-cigarette smoking, cannabis use, problematic Internet use) that a person displays. Secondary outcomes are metric measures reflecting (1) alcohol use, (2) tobacco/e-cigarette use, (3) cannabis use, (4) problematic Internet use as well as (5) general self-efficacy [36] and (6) stress [37].

### Data analyses

Generalized Linear Mixed Models will be used to test intervention effects for binary and count outcomes and Linear Mixed Models for continuous outcomes [38, 39]. These models account for both fixed and random effects and are particularly useful in analysing longitudinal and nested data (e.g., students within school classes). To test the efficacy of the intervention, we will test the variables “study group”, “time” and their interaction “study group x time” as predictors of the outcome criteria assessed at follow-up. If necessary, we will control baseline differences by adding additional baseline variables as covariates to the models. We will conduct both (1) intention to treat analyses and (2) complete case analyses considering all study participants with available follow-up data. For ITT analyses, we will use multiple imputation procedures as described elsewhere [40].

## Discussion

Entering work life, typically in late adolescence, is associated with mutual challenges and health risks. *ready4life* is the first mHealth program for addiction prevention in apprentices that will be tested within a controlled trial. The program takes into account the heterogeneity of adolescent addictive behaviour by promoting life skills on the one hand and reducing risk behaviours on the other. Addressing four major addictive behaviours among adolescents [8] as well as two life-skills does not only have the potential to reduce substance and internet use in the short term, but may also lead to increased general self-efficacy and life skills, such as coping with stress, which in the long term might help to prevent further non-communicable diseases and improve well-being [41, 42]. In contrast to comprehensive school-based curricula, training and counselling via the *ready4life* smartphone app is more economic and corresponds with the lifestyle and communication habits of the target group. A majority of apprentices are familiar with how to use smartphones and typically use them on a daily basis for texting, taking photos, playing games etc. Nonetheless, continued use and adherence to eHealth or mHealth intervention programs presents a serious challenge [43, 44]. Although, we implemented several measures like push notifications, gamification elements like quizzes, the collection of credits for program engagement or a price draw, we are aware that the app will compete with many others. Therefore, besides program efficacy, the investigation of program engagement and its association with program outcome presents another focus of this research project [43, 45].

The presented efficacy study on this program is of high scientific and public health relevance. It will reveal whether this universally implementable but individually tailored intervention approach is effective in increasing life skills and reducing risk behaviours in a group of adolescents with a particular high risk of addictive behaviours [7]. If this program proves to be effective, it could be disseminated to apprentices in different settings, e.g., schools, companies or via online platforms for apprentices or vocational students. Furthermore, an adaption for youth in general or the translation of the intervention content into other languages would easily enable program dissemination.

## Data Availability

The datasets generated during the current study are available from the corresponding author on reasonable request.
